# An ectopic adrenocortical adenoma in renal hilum presenting with Cushing's syndrome: A case report and literature review

**DOI:** 10.1097/MD.0000000000013322

**Published:** 2018-12-14

**Authors:** Difei Lu, Nan Yu, Xiaowei Ma, Junqing Zhang, Xiaohui Guo

**Affiliations:** Department of Endocrinology, Peking University First Hospital, China.

**Keywords:** Cushing's syndrome, ectopic adrenocortical adenoma, renal hilum

## Abstract

**Rationale:**

Ectopic adrenal tissue is the adrenal rests along the path from gonads to adrenal glands during embryogenesis. Ectopic adrenocortical adenoma is a rare disease represented with over-production of cortisol by the ectopic adrenocortical tissue.

**Patient concerns:**

An 18-year-old Chinese female patient was presented with weight-gain for 6 months. She had elevated plasma cortisol and a solitary mass was revealed using computed tomography scan in the left renal hilum.

**Diagnosis:**

The tumor was removed and the immunohistochemical profile indicated an ectopic adrenocortical adenoma.

**Interventions:**

After the tumor was removed, the patient was under glucocorticoid replacement therapy in 6-month.

**Outcomes:**

During 6-month of follow-up, the patient showed no signs of tumor recurrence.

**Lessons:**

Ectopic adrenocortical adenoma is difficult to diagnose due to its low incidence, and the ectopic rests in renal hilum could be misdiagnosed as renal cell carcinoma. This case reminds clinicians to be aware of ectopic site in the diagnosis of adrenocorticotropic hormone (ACTH) independent Cushing's syndrome. Immunohistochemical stain may assist in evaluating the origin of the ectopic rests. A certain rate of local recurrence indicated the need of long-term follow-up.

## 1 Introduction

The adrenal glands are of a dual embryological origin. The adrenal cortex is derived form the coelomic mesoderm of the urogenital ridge, and the adrenal medulla arises form neural crest tissue.^[1]^ Ectopic adrenocortical rests consist of adrenal cortex tissue since no reports of medullary tissue were reported. Ectopic adrenal rests exists along the migration path of adrenal cortex development, and these anatomic sites includes celiac plexus, kidney, broad ligament, testis, and spermatic cord.^[2,3]^ The hormone secreting status decides the clinical features of ectopic adrenal rests, and those functional rests are symptomatic and more detectable than non-functional ones. Here, we report a case of ectopic adrenocortical adenoma located in the left hilum.

## 2 Case presentation

**Figure 1:**
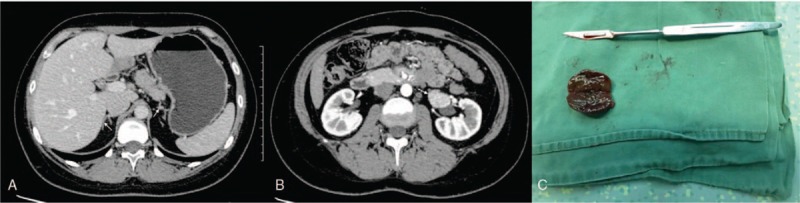
A: Abdominal CT of the patient. Enhanced CT showed a well-circumscribed round mass with a maximum diameter of 3.0 cm in the left renal hilum. B: CT scan revealed bilateral adrenal glands were atrophic. 1C: the ectopic adrenal tumor was resected during the operation, and was sized 3.0cm × 2.5cm × 1.5 cm. CT = computed tomography.

An 18-year-old Chinese female patient complained of faciotruncal obesity of 6 months duration, accompanied with irregular menstruation, easy bruising, facial acne and purple striae on her legs. Before she was admitted to our hospital on June 14, 2017, she had amenorrhea for 2 months. Her medical history was unremarkable. During hospitalization, her blood pressure (BP) and blood glucose remained normal (BP: 134/88mmHg, fasting blood glucose 4.8mmol/L). Her body mass index was 26.6 kg/m^2^. Plasma cortisol concentrations were 20.67 μg/dL in the morning [8am, 571.3nmol/L, normal: 4.4-19.9 μg/dL (121.6-550.0 nmol/L)], 17.67 μg/dL in the afternoon (4pm, 488.7nmol/L) and 18.95 μg/dL in the midnight (0am, 523.8nmol/L), indicated that the normal circadian rhythm was lost. The morning, afternoon and midnight plasma adrenocorticotropic hormone (ACTH) concentration was 1.32 pg/mL (normal: 7.2–63.3pg/mL), 1.37pg/mL, and 1.01pg/mL, respectively. Urinary free cortisol (UFC) concentration was 1824 μg/24 h (normal: 100-379 μg/24 h). During the low dose dexamethasone depression test (LDDST), the morning plasma cortisol slightly increased from 20.67 μg/dL to 25.66 μg/dL, and the UFC after oral dexamethasone was 1388.8 μg/24 h. The patient underwent a high dose dexamethasone suppression test (HDDST), the morning plasma cortisol was 27.52 μg/dL and UFC was 1726.1 μg/24 h after oral dexamethasone intervention. Both morning plasma cortisol and UFC in LDDST and HDDST were not suppressed, supporting the diagnosis of ACTH-independent Cushing's syndrome. Adrenal computed tomography (CT) scan revealed a well-circumscribed round mass with a maximum diameter of 3.0 cm in the left renal hilum, and bilateral adrenal glands were atrophic (Fig. 1A, Fig. 1B). The tumor was clinically suspected as ectopic adrenal cortical adenoma. Ultrasound of obstetrics and gynecology was performed to rule out other possible ectopic adrenal rests, and the ultrasound indicated polycystic ovaries (over 12 cysts for each ovary).

The patient underwent laparoscopic resection of the tumor. During the operation, the mass in the left renal hilum was completely resected. Postoperative pathology results confirmed the diagnosis of ectopic adrenocortical adenoma. The patient was followed up for 12 months after the operation. She was under glucocorticoid replacement therapy for 8 months, and hydrocortisone 100 mg was intravenously given in the operating day and 2 days after the operation. Oral prednisone (10 mg bid) was given 7 days after the operation for a week and gradually tapered for 8 months duration according to clinical symptoms and morning plasma cortisol. No tumor recurrence or metastasis was found after 12 months of follow-up.

### 2.1 Gross features

The tumor was in size of 3.0cm × 2.5cm × 1.5 cm. Grossly, the mass was round, solid and well circumscribed. Its cut surface was brown (Fig. 1C).

### 2.2 Immunohistochemistry

**Figure 2:**
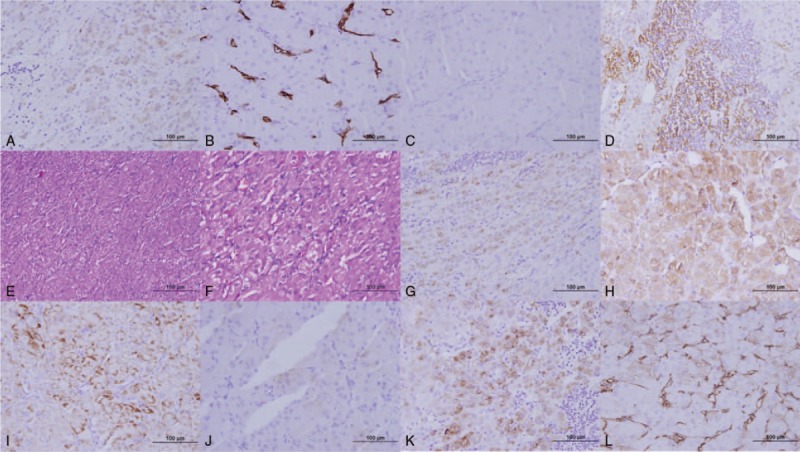
The immunohistological stain of the ectopic adrenal tumor. 2A: AE1/AE3 (+). 2B: angiographic positive for CD34. 2C: CgA (−). 2D: CgA-OR (+). 2E and 2F: HE stain. 2G: partially positive for HMB45. 2H: inhibitin (+++). 2L: Melan-A (+). 2J: NSE (−). 2K: Synaptophysin (+). 2L: vimentin (+).

Immunohistochemistry stain showed the tissue was positive for inhibition, Melan-A, synaptophysin, vimentin and AE1/AE3, partially positive for HMB45, angiographic positive for CD34 and negative for NSE and CgA (Fig. 2).

## 3 Discussion

Adrenal rest tissue is formed during development when adrenal tissues break off and fragments stay in the path of migration. Ectopic adrenal tissue is reported in 50% neonates, and most of the ectopic rests will atrophy.^[4]^ The occurrence of adrenal rest tissue in adults is 1%.^[5]^ The fetal adrenal cortex is formed during the 5th week of fetal development, when mesothelial cells from the posterior abdominal wall develop urogenital ridge and separates from the rest mesothelial cells by 8th week.^[6]^ In early embryogenesis, adrenal cortex ascends from the urogenital ridge, so are gonads. Adrenal rests are discovered in migrating path descending with gonads, including in the retroperitoneum, testis, broad ligament, kidneys, ovaries and inguinal region.^[7]^ Brain, lung and stomach are also reported to be rare sites of ectopic rests.^[2,3]^

After regression of the adrenal rests, they are incidentally found in surgery or autopsy and are non-symptomatic. When hyperplastic change happen to the adrenal rests and subsequently result in over-production of cortisol, they are of clinical importance. Excess plasma cortisol can lead to Cushing's syndrome, and clinical symptoms involve amenorrhea, obesity, easy bruising. Here, we made a literature review of ectopic adrenal adenoma in renal hilum, reported in English language literature (Table 1).

**Table 1:**
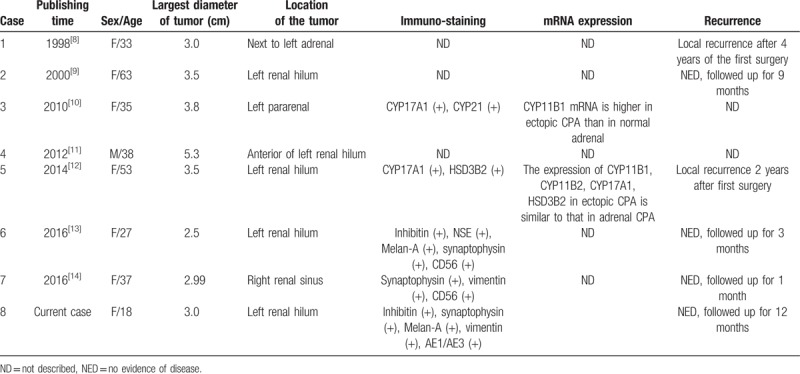
Case reports of ectopic adrenocortical adenoma in renal hilum.

CT scan is a sensitive method to locate the ectopic adrenal tumor. However, it is a challenge to confirm the hormone-producing function of the tumor before surgery. Imaging characteristics of ectopic adrenocortical adenoma could assist clinical diagnosis. In our case, cortisol circadian rhythm, LDDST, and HDDST indicated ACTH-independent Cushing's syndrome, and CT showed bilateral adrenal atrophied, thus an ectopic adrenocortical adenoma was suspected. The tumor in CT was a solid, well-circumscribed one, and heterogeneously enhanced in arterial phase, indicating a benign tumor. The benign tumors in perirenal space should be distinguished from pheochromocytoma, leiomyoma or lymphoma, which is of difficulty using only CT. The immunohistochemistry stain of the tumor (inhibitin +, Melan-A +, synaptophysin +, vimentin + and AE1/AE3+) supported an adrenocortical origin rather than a renal or mesenchymal origin, which was in accordance with preview reports.^[13,14]^ Therefore, a pre-operative biopsy of the tumor, if possible, should be performed to confirm the diagnosis of ectopic adrenocortical adenoma and provide evidence for clinical treatment.

The differential diagnosis from adrenocortical adenocarcinoma is of great importance. In our case, the imaging of the tumor was not as large in diameter as most adrenocortical adenocarcinomas and showed no features of malignancy, including capsular or vascular invasion, necrosis, and distant metastasis. Histological stain of the tumor could also evaluate the malignancy of adrenocortical tumors. Histological signs of malignancy include necrosis, abnormal mitosis and capsular invasion, as was summarized in Weiss criteria.^[15]^ The confirmation of malignancy depends on tumor behavior of malignancy, such as distant metastasis and invasion, which suggests the critical role of post-operative follow-up.

The treatment of functioning ectopic adrenal tumors is radical resection. Otherwise, watchful waiting is a choice for nonfunctioning ones. After surgery, long-term follow-up is of great importance due to the possibility of recurrence. In the previous reported cases, 2 patients appeared symptoms of Cushing's syndrome, and local recurrence of adrenocortical adenoma was diagnosed.^[8,12]^ Other ectopic adrenal rests were not found clinical evidence of recurrence, while the duration of follow-up was no long enough for some of the cases. In Cushing's disease and adrenocortical adenoma, there is possibility of recurrence and long-term follow-up is recommended.^[16]^ For ectopic adrenocortical adenoma, long-term follow-up consists of surveillance of morning plasma cortisol and imaging examination, such as CT, to rule out local recurrence.

## 4 Conclusions

This case provides an extremely rare case of ectopic adrenocortical adenoma in the left renal hilum to clinicians as a reminder of the scarce situation. Pre-operative biopsy and immunohistochemical staining could help in diagnosis and confirming the origin of the tumor. Long-term follow-up is necessary in both plasma cortisol and imaging examination due to the possibility of local recurrence.

## Acknowledgment

We thank the patient who agreed to our report with anonymity and for providing a detailed medical history.

## Author contributions

**Data curation:** Difei Lu.

**Formal analysis:** Difei Lu.

**Investigation:** Difei Lu.

**Methodology:** Nan Yu.

**Supervision:** Nan Yu.

**Validation:** Difei Lu.

**Visualization:** Difei Lu.

**Writing – original draft:** Difei Lu.

**Writing – review & editing:** Nan Yu, Xiaowei Ma, Xiaohui Guo, Junqing Zhang.

Difei Lu orcid: 0000-0003-3953-8900.
